# Supplementary material for: Both consumptive and non-consumptive effects of predators impact mosquito populations and have implications for disease transmission
Source: eLife. 2022 Jan 19;11:e71503. doi: 10.7554/eLife.71503 (PMC8769645; doi:10.7554/eLife.71503)
Supplement: Supplementary file 1. [file elife-71503-supp1.docx]

Table 1: Publications included in the database and their types of vector trait data

| **Publication** | **Vector Trait** | | | |
| --- | --- | --- | --- | --- |
|  | Survival | Behavior | Size | Development |
| (Alcalay et al., 2018) |  |  |  |  |
| (Alcalay et al., 2019) |  |  |  |  |
| (Arav and Blaustein, 2006) |  |  |  |  |
| (Beketov and Liess, 2007) |  |  |  |  |
| (Blaustein et al., 2005) |  |  |  |  |
| (Blaustein and Margalit, 1996) |  |  |  |  |
| (Bond et al., 2005) |  |  |  |  |
| (Bowatte et al., 2013) |  |  |  |  |
| (Bucciarelli et al., 2019) |  |  |  |  |
| (Calliari et al., 2003) |  |  |  |  |
| (Chandrasegaran et al., 2018) |  |  |  |  |
| (Chansang et al., 2004) |  |  |  |  |
| (Cuthbert et al., 2018) |  |  |  |  |
| (Cuthbert et al., 2019a) |  |  |  |  |
| (Cuthbert et al., 2019b) |  |  |  |  |
| (de Valdez, 2007) |  |  |  |  |
| (Dieng et al., 2002) |  |  |  |  |
| (Dieng et al., 2003) |  |  |  |  |
| (Farajollahi et al., 2009) |  |  |  |  |
| (Fischer et al., 2012) |  |  |  |  |
| (Fontana-Bria et al., 2017) |  |  |  |  |
| (Futami et al., 2008) |  |  |  |  |
| (Grill and Juliano, 1996) |  |  |  |  |
| (Griswold and Lounibos, 2005) |  |  |  |  |
| (Irwin and Paskewitz, 2009) |  |  |  |  |
| (Kalimuthu et al., 2017) |  |  |  |  |
| (Knight et al., 2004) |  |  |  |  |
| (Kraus and Vonesh, 2010) |  |  |  |  |
| (Krol et al., 2019) |  |  |  |  |
| (Kweka et al., 2011) |  |  |  |  |
| (Marten et al., 2000a) |  |  |  |  |
| (Marten et al., 2000b) |  |  |  |  |
| (Mercer et al., 2005) |  |  |  |  |
| (Micieli et al., 2002) |  |  |  |  |
| (Minakawa et al., 2007) |  |  |  |  |
| (Nannini and Juliano, 1998) |  |  |  |  |
| (Ohba et al., 2012) |  |  |  |  |
| (Op de Beeck et al., 2016) |  |  |  |  |
| (Orr and Resh, 1989) |  |  |  |  |
| (Pintar et al., 2018) |  |  |  |  |
| (Roberts, 2012) |  |  |  |  |
| (Roberts, 2018) |  |  |  |  |
| (Roux et al., 2015) |  |  |  |  |

| **Publication (continued)** | **Vector Trait (continued)** | | | |
| --- | --- | --- | --- | --- |
|  | Survival | Behavior | Size | Development |
| (Rubbo et al., 2011) |  |  |  |  |
| (Schrama et al., 2018) |  |  |  |  |
| (Silberbush et al., 2015) |  |  |  |  |
| (Silberbush and Blaustein, 2008) |  |  |  |  |
| (Silberbush and Blaustein, 2011) |  |  |  |  |
| (Soghigian et al., 2017) |  |  |  |  |
| (Staats et al., 2016) |  |  |  |  |
| (Stav et al., 2005) |  |  |  |  |
| (Subramaniam et al., 2015) |  |  |  |  |
| (Tietze and Mulla, 1990) |  |  |  |  |
| (van Uitregt et al., 2012) |  |  |  |  |
| (van Uitregt et al., 2013) |  |  |  |  |
| (Veronesi et al., 2015) |  |  |  |  |
| (Wallace and Merritt, 1999) |  |  |  |  |
| (Yanoviak, 1999) |  |  |  |  |
| (Zuharah et al., 2013) |  |  |  |  |
| (Zuharah and Lester, 2010) |  |  |  |  |

**References**

ALCALAY, Y., TSURIM, I. & OVADIA, O. 2018. Female mosquitoes disperse further when they develop under predation risk. *Behavioral Ecology,* 29**,** 1402-1408.

ALCALAY, Y., TSURIM, I. & OVADIA, O. 2019. Multi-scale oviposition site selection in two mosquito species. *Ecological Entomology,* 44**,** 347-356.

ARAV, D. & BLAUSTEIN, L. 2006. Effects of pool depth and risk of predation on oviposition habitat selection by temporary pool dipterans. *Journal of Medical Entomology,* 43**,** 493-497.

BEKETOV, M. A. & LIESS, M. 2007. Predation risk perception and food scarcity induce alterations of life-cycle traits of the mosquito Culex pipens. *Ecological Entomology,* 32**,** 405-410.

BLAUSTEIN, L., BLAUSTEIN, J. & CHASE, J. 2005. Chemical detection of the predator Notonecta irrorata by ovipositing Culex mosquitoes. *Journal of Vector Ecology,* 30**,** 299-301.

BLAUSTEIN, L. & MARGALIT, J. 1996. Priority effects in temporary pools: Nature and outcome of mosquito larva-toad tadpole interactions depend on order of entrance. *Journal of Animal Ecology,* 65**,** 77-84.

BOND, J. G., ARREDONDO-JIMENEZ, J. I., RODRIGUEZ, M. H., QUIROZ-MARTINEZ, H. & WILLIAMS, T. 2005. Oviposition habitat selection for a predator refuge and food source in a mosquito. *Ecological Entomology,* 30**,** 255-263.

BOWATTE, G., PERERA, P., SENEVIRATHNE, G., MEEGASKUMBURA, S. & MEEGASKUMBURA, M. 2013. Tadpoles as dengue mosquito (Aedes aegypti) egg predators. *Biological Control,* 67**,** 469-474.

BUCCIARELLI, G. M., SUH, D., LAMB, A. D., ROBERTS, D., SHARPTON, D., SHAFFER, H. B., FISHER, R. N. & KATS, L. B. 2019. Assessing effects of non-native crayfish on mosquito survival. *Conservation Biology,* 33**,** 122-131.

CALLIARI, D., SANZ, K., MARTINEZ, M., CERVETTO, G., GOMEZ, M. & BASSO, C. 2003. Comparison of the predation rate of freshwater cyclopoid copepod species on larvae of the mosquito Culex pipiens. *Medical and veterinary entomology,* 17**,** 339-342.

CHANDRASEGARAN, K., SINGH, A., LAHA, M. & QUADER, S. 2018. Playing it safe? Behavioural responses of mosquito larvae encountering a fish predator. *Ethology Ecology & Evolution,* 30**,** 70-87.

CHANSANG, U. R., BHUMIRATANA, A. & KITTAYAPONG, P. 2004. Combination of Mesocyclops thermocyclopoides and Bacillus thuringiensis var. israelensis: A better approach for the control of Aedes aegypti larvae in water containers. *Journal of Vector Ecology,* 29**,** 218-226.

CUTHBERT, R. N., DALU, T., WASSERMAN, R. J., COUGHLAN, N. E., CALLAGHAN, A., WEYL, O. L. F. & DICK, J. T. A. 2018. Muddy waters: Efficacious predation of container-breeding mosquitoes by a newly-described calanoid copepod across differential water clarities. *Biological Control,* 127**,** 25-30.

CUTHBERT, R. N., DALU, T., WASSERMAN, R. J., WEYL, O. L. F., CALLAGHAN, A., FRONEMAN, W. & DICK, J. T. A. 2019a. Sex-skewed trophic impacts in ephemeral wetlands. *Freshwater Biology,* 64**,** 359-366.

CUTHBERT, R. N., ORTIZ-PEREA, N., DICK, J. T. A. & CALLAGHAN, A. 2019b. Elusive enemies: Consumptive and ovipositional effects on mosquitoes by predatory midge larvae are enhanced in dyed environments. *Biological Control,* 132**,** 116-121.

DE VALDEZ, M. R. 2007. Predator avoidance behavior of Aedes aegypti mosquito larvae infected with mermithid nematodes (Nematoda: Mermithidae). *J Vector Ecol,* 32**,** 150-3.

DIENG, H., BOOTS, M., TUNO, N., TSUDA, Y. & TAKAGI, M. 2002. A laboratory and field evaluation of Macrocyclops distinctus, Megacyclops viridis and Mesocyclops pehpeiensis as control agents of the dengue vector Aedes albopictus in a peridomestic area in Nagasaki, Japan. *Medical and Veterinary Entomology,* 16**,** 285-291.

DIENG, H., BOOTS, M., TUNO, N., TSUDA, Y. & TAKAGI, M. 2003. Life history effects of prey choice by copepods: Implications for biocontrol of vector mosquitoes. *Journal of the American Mosquito Control Association,* 19**,** 67-73.

FARAJOLLAHI, A., KESAVARAJU, B., NELDER, M. P., CRANS, S. C. & GAUGLER, R. 2009. An unusual larval collection and survival of Orthopodomyia signifera in the presence of the predator Toxorhynchites rutilus septentrionalis. *J Am Mosq Control Assoc,* 25**,** 370-3.

FISCHER, S., PEREYRA, D. & FERNÁNDEZ, L. 2012. Predation ability and non-consumptive effects of Notonecta sellata (Heteroptera: Notonectidae) on immature stages of Culex pipiens (Diptera: Culicidae). *Journal of Vector Ecology,* 37**,** 245-251.

FONTANA-BRIA, L., SELFA, J., TUR, C. & FRAGO, E. 2017. Early exposure to predation risk carries over metamorphosis in two distantly related freshwater insects. *Ecological Entomology,* 42**,** 255-262.

FUTAMI, K., SONYE, G., AKWEYWA, P., KANEKO, S. & MINAKAWA, N. 2008. Diving behavior in Anopheles gambiae (Diptera: Culicidae): avoidance of a predacious wolf spider (Araneae: Lycosidae) in relation to life stage and water depth. *J Med Entomol,* 45**,** 1050-6.

GRILL, C. P. & JULIANO, S. A. 1996. Predicting species interactions based on behaviour: Predation and competition in container-dwelling mosquitoes. *Journal of Animal Ecology,* 65**,** 63-76.

GRISWOLD, M. W. & LOUNIBOS, L. P. 2005. Competitive outcomes of aquatic container Diptera depend on predation and resource levels. *Annals of the Entomological Society of America,* 98**,** 673-681.

IRWIN, P. & PASKEWITZ, S. 2009. Investigation of fathead minnows (Pimephales promelas) as a biological control agent of Culex mosquitoes under laboratory and field conditions. *J Am Mosq Control Assoc,* 25**,** 301-9.

KALIMUTHU, K., PANNEERSELVAM, D. C., CHOU, C., LIN, S.-M., TSENG, L.-C., TSAI, K.-H., MURUGAN, K. & HWANG, J.-S. 2017. Predatory efficiency of the copepod Megacyclops formosanus and toxic effect of the red alga Gracilaria firma-synthesized silver nanoparticles against the dengue vector Aedes aegypti. *Hydrobiologia,* 785.

KNIGHT, T. M., CHASE, J. M., GOSS, C. W. & KNIGHT, J. J. 2004. Effects of interspecific competition, predation, and their interaction on survival and development time of immature Anopheles quadrimaculatus. *J Vector Ecol,* 29**,** 277-84.

KRAUS, J. M. & VONESH, J. R. 2010. Feedbacks between community assembly and habitat selection shape variation in local colonization. *Journal of Animal Ecology,* 79**,** 795-802.

KROL, L., GORSICH, E. E., HUNTING, E. R., GOVENDER, D., VAN BODEGOM, P. M. & SCHRAMA, M. 2019. Eutrophication governs predator-prey interactions and temperature effects in Aedes aegypti populations. *Parasites & Vectors,* 12.

KWEKA, E. J., ZHOU, G. F., GILBREATH, T. M., AFRANE, Y., NYINDO, M., GITHEKO, A. K. & YAN, G. Y. 2011. Predation efficiency of Anopheles gambiae larvae by aquatic predators in western Kenya highlands. *Parasites & Vectors,* 4.

MARTEN, G. G., NGUYEN, M., MASON, B. J. & NGO, G. 2000a. Natural control of Culex quinquefasciatus larvae in residential ditches by the copepod Macrocyclops albidus. *Journal of Vector Ecology,* 25**,** 7-15.

MARTEN, G. G., NGUYEN, M. & NGO, G. 2000b. Copepod predation on Anopheles quadrimaculatus larvae in rice fields. *Journal of Vector Ecology,* 25**,** 1-6.

MERCER, D. R., WETTACH, G. R. & SMITH, J. L. 2005. Effects of larval density and predation by Toxorhynchites amboinensis on Aedes polynesiensis (Diptera : culicidae) developing in coconuts. *Journal of the American Mosquito Control Association,* 21**,** 425-431.

MICIELI, M. V., MARTI, G. & GARCÍA, J. J. 2002. Laboratory evaluation of Mesocyclops annulatus (Wierzejski, 1892) (Copepoda: Cyclopidea) as a predator of container-breeding mosquitoes in Argentina. *Mem Inst Oswaldo Cruz,* 97**,** 835-8.

MINAKAWA, N., FUTAMI, K., SONYE, G., AKWEYWA, P. & KANEKO, S. 2007. Predatory capacity of a shorefly, Ochthera chalybescens, on malaria vectors. *Malaria Journal,* 6**,** 104.

NANNINI, M. A. & JULIANO, S. A. 1998. Effects of the facultative predator Anopheles barberi on population performance of its prey Aedes triseriatus (Diptera Culicidae). *Annals of the Entomological Society of America,* 91**,** 33-42.

OHBA, S.-Y., OHTSUKA, M., SUNAHARA, T., SONODA, Y., KAWASHIMA, E. & TAKAGI, M. 2012. Differential responses to predator cues between two mosquito species breeding in different habitats. *Ecological Entomology,* 37**,** 410-418.

OP DE BEECK, L., JANSSENS, L. & STOKS, R. 2016. Synthetic predator cues impair immune function and make the biological pesticide Bti more lethal for vector mosquitoes. *Ecological Applications,* 26**,** 355-366.

ORR, B. K. & RESH, V. H. 1989. Experimental test of the influence of aquatic macrophyte cover on the survival of Anopheles larvae. *J Am Mosq Control Assoc,* 5**,** 579-85.

PINTAR, M. R., BOHENEK, J. R., EVELAND, L. L. & RESETARITS, W. J. 2018. Colonization across gradients of risk and reward: Nutrients and predators generate species-specific responses among aquatic insects. *Functional Ecology,* 32**,** 1589-1598.

ROBERTS, D. 2012. Responses of three species of mosquito larvae to the presence of predatory dragonfly and damselfly larvae. *Entomologia Experimentalis Et Applicata,* 145**,** 23-29.

ROBERTS, D. 2018. Predator feeding vibrations encourage mosquito larvae to shorten their development and so become smaller adults: Predator feeding vibrations. *Ecological Entomology*.

ROUX, O., VANTAUX, A., ROCHE, B., YAMEOGO, K. B., DABIRE, K. R., DIABATE, A., SIMARD, F. & LEFEVRE, T. 2015. Evidence for carry-over effects of predator exposure on pathogen transmission potential. *Proceedings of the Royal Society B-Biological Sciences,* 282**,** 20152430.

RUBBO, M. J., LANTERMAN, J. L., FALCO, R. C. & DANIELS, T. J. 2011. The influence of amphibians on mosquitoes in seasonal pools: Can wetlands protection help to minimize disease risk? *Wetlands,* 31**,** 799-804.

SCHRAMA, M., GORSICH, E. E., HUNTING, E., BARMENTLO, S. H., BEECHLER, B. & VAN BODEGOM, P. M. 2018. Eutrophication and predator presence overrule the effects of temperature on mosquito survival and development. *PLoS Neglected Tropical Diseases,* 3.

SILBERBUSH, A., ABRAMSKY, Z. & TSURIM, I. 2015. Effects of fish cues on mosquito larvae development. *Acta Tropica,* 150**,** 196-199.

SILBERBUSH, A. & BLAUSTEIN, L. 2008. Oviposition habitat selection by a mosquito in response to a predator: Are predator-released kairomones air-borne cues? *J Vector Ecol,* 33**,** 208-11.

SILBERBUSH, A. & BLAUSTEIN, L. 2011. Mosquito females quantify risk of predation to their progeny when selecting an oviposition site. *Functional Ecology,* 25**,** 1091-1095.

SOGHIGIAN, J., VALSDOTTIR, L. R. & LIVDAHL, T. P. 2017. A parasite's modification of host behavior reduces predation on its host. *Ecology and Evolution,* 7**,** 1453-1461.

STAATS, E. G., AGOSTA, S. J. & VONESH, J. R. 2016. Predator diversity reduces habitat colonization by mosquitoes and midges. *Biology Letters,* 12**,** 20160580.

STAV, G., BLAUSTEIN, L. & MARGALIT, Y. 2005. Individual and interactive effects of predator and controphic species on mosquito populations. *Ecological Applications,* 15**,** 587-598.

SUBRAMANIAM, J., MURUGAN, K., PANNEERSELVAM, C., KOVENDAN, K., MADHIYAZHAGAN, P., KUMAR, P. M., DINESH, D., CHANDRAMOHAN, B., SURESH, U., NICOLETTI, M., HIGUCHI, A., HWANG, J. S., KUMAR, S., ALARFAJ, A. A., MUNUSAMY, M. A., MESSING, R. H. & BENELLI, G. 2015. Eco-friendly control of malaria and arbovirus vectors using the mosquitofish Gambusia affinis and ultra-low dosages of Mimusops elengi-synthesized silver nanoparticles: towards an integrative approach? *Environ Sci Pollut Res Int,* 22**,** 20067-83.

TIETZE, N. S. & MULLA, M. S. 1990. Influence of tadpole shrimp, Triops longicaudatus (Notostraca:Triopsidae), stocking rate on Culex tarsalis development in experimental field microcosms. *J Am Mosq Control Assoc,* 6**,** 265-9.

VAN UITREGT, V. O., HURST, T. P. & WILSON, R. S. 2012. Reduced size and starvation resistance in adult mosquitoes, Aedes notoscriptus, exposed to predation cues as larvae. *Journal of Animal Ecology,* 81**,** 108-115.

VAN UITREGT, V. O., HURST, T. P. & WILSON, R. S. 2013. Greater costs of inducible behavioural defences at cooler temperatures in larvae of the mosquito, Aedes notoscriptus. *Evolutionary Ecology,* 27**,** 13-26.

VERONESI, R., CARRIERI, M., MACCAGNANI, B., MAINI, S. & BELLINI, R. 2015. Macrocyclops albidus (Copepoda: Cyclopidae) for the biocontrol of Aedes albopictus and Culex pipiens in Italy. *Journal of the American Mosquito Control Association,* 31**,** 32-43.

WALLACE, J. R. & MERRITT, R. W. 1999. Influence of microclimate, food, and predation on Anopheles quadrimaculatus (Diptera : Culicidae) growth and development rates, survivorship, and adult size in a Michigan pond. *Environmental Entomology,* 28**,** 233-239.

YANOVIAK, S. P. 1999. Effects of Mecistogaster spp. (Odonata: Pseudostigmatidae) and Culex mollis (Diptera: Culicidae) on litter decomposition in neotropical treehole microcosms. *The Florida Entomologist,* 82**,** 462-468.

ZUHARAH, W. F., FADZLY, N. & LESTER, P. J. 2013. Lethal and sublethal impacts of predaceous backswimmer Anisops wakefieldi (Hemiptera: Notonectidae) on the life-history traits of the New Zealand mosquito Culex pervigilans (Diptera: Culicidae). *J Med Entomol,* 50**,** 1014-24.

ZUHARAH, W. F. & LESTER, P. J. 2010. The influence of aquatic predators on mosquito abundance in animal drinking troughs in New Zealand. *Journal of Vector Ecology,* 35**,** 347-353.
