# Supplementary material for: Both consumptive and non-consumptive effects of predators impact mosquito populations and have implications for disease transmission
Source: eLife. 2022 Jan 19;11:e71503. doi: 10.7554/eLife.71503 (PMC8769645; doi:10.7554/eLife.71503)
Supplement: Transparent reporting form [file elife-71503-transrepform1.pdf]

eLIFE
1st Floor
24 Hills Road
Cambridge CB2 1JP, UK
P 01223 855340
W elifesciences.org
T @elife

eLife's transparent reporting form

We encourage authors to provide detailed information *within their submission* to facilitate the interpretation and replication of experiments. Authors can upload supporting documentation to indicate the use of appropriate reporting guidelines for health-related research (see EQUATOR Network), life science research (see the BioSharing Information Resource), or the ARRIVE guidelines for reporting work involving animal research. Where applicable, authors should refer to any relevant reporting standards documents in this form.

If you have any questions, please consult our Journal Policies and/or contact us: editorial@elifesciences.org.

Sample-size estimation

- You should state whether an appropriate sample size was computed when the study was being designed
- You should state the statistical method of sample size computation and any required assumptions
- If no explicit power analysis was used, you should describe how you decided what sample (replicate) size (number) to use

Please outline where this information can be found within the submission (e.g., sections or figure legends), or explain why this information doesn't apply to your submission:

The sample sizes that we worked with in this study were influenced by what was available in the existing literature. The process that we followed to collect observations from past studies is described in the "Materials and Methods" section, and depicted in Figure 1. We explain how sample size constraints guided the type of analyses performed in the "Data Analysis: Testing Moderators" sub-section.

Replicates

- You should report how often each experiment was performed
- You should include a definition of biological versus technical replication
- The data obtained should be provided and sufficient information should be provided to indicate the number of independent biological and/or technical replicates
- If you encountered any outliers, you should describe how these were handled
- Criteria for exclusion/inclusion of data should be clearly stated
- High-throughput sequence data should be uploaded before submission, with a private link for reviewers provided (these are available from both GEO and ArrayExpress)

Please outline where this information can be found within the submission (e.g., sections or figure legends), or explain why this information doesn't apply to your submission:

In our database, which is privately available to reviewers and will eventually become public, we include both a "Pred_n" column that lists the number of replicates in the predator treatment, and a "Con_n" column that lists the number of replicates in the control, for each of our 568 observations. In the context of predation assays, replication is biological rather than technical because advanced equipment that could be sensitive to noise is rarely used in these types of experiments. Criteria for exclusion/inclusion of data are explained in the "Materials and Methods" section and summarized by the flowchart in Figure 1. Here is a private link to our database: https://datadryad.org/stash/share/DhlrrGPTDUS4LMQF8--vP7PBHhcExyF2HQlJOixSOno

Statistical reporting

- Statistical analysis methods should be described and justified
- Raw data should be presented in figures whenever informative to do so (typically when N per group is less than 10)
- For each experiment, you should identify the statistical tests used, exact values of N, definitions of center, methods of multiple test correction, and dispersion and precision measures (e.g., mean, median, SD, SEM, confidence intervals; and, for the major substantive results, a measure of effect size (e.g., Pearson's r, Cohen's d)
- Report exact p-values wherever possible alongside the summary statistics and 95% confidence intervals. These should be reported for all key questions and not only when the p-value is less than 0.05.

Please outline where this information can be found within the submission (e.g., sections or figure legends), or explain why this information doesn't apply to your submission:

Statistical analysis methods are described and justified in the "Data Analysis" sub-section of the "Materials and Methods" section. Exact p-values are included alongside every 95% confidence interval reported in the "Results" section.

(For large datasets, or papers with a very large number of statistical tests, you may upload a single table file with tests, Ns, etc., with reference to sections in the manuscript.)

Group allocation

- Indicate how samples were allocated into experimental groups (in the case of clinical studies, please specify allocation to treatment method); if randomization was used, please also state if restricted randomization was applied
- Indicate if masking was used during group allocation, data collection and/or data analysis

Please outline where this information can be found within the submission (e.g., sections or figure legends), or explain why this information doesn't apply to your submission:

Three hundred twenty observations from our database were separated into seven different data subsets for analysis. These subsets were determined by the vector trait that was measured, and whether the effect of the predator(s) on the trait was consumptive or non-consumptive. The subsets are shown in Figure 1.

Additional data files ("source data")

- We encourage you to upload relevant additional data files, such as numerical data that are represented as a graph in a figure, or as a summary table
- Where provided, these should be in the most useful format, and they can be uploaded as "Source data" files linked to a main figure or table
- Include model definition files including the full list of parameters used
- Include code used for data analysis (e.g., R, MatLab)
- Avoid stating that data files are "available upon request"

Please indicate the figures or tables for which source data files have been provided:

eLife Sciences Publications, Ltd is a limited liability non-profit non-stock corporation incorporated in the State of Delaware, USA, with company number 5030732, and is registered in the UK with company number FC030576 and branch number BR015634 at the address 1st Floor, 24 Hills Road, Cambridge CB2 1JP | August 2014

Our database and R code file, showing all meta-analyses, are available for private review: https://datadryad.org/stash/share/DhlrrGPTDUS4LMQF8--vP7PBHhcExyF2HQlJOixSOno
These are the source data files for Figures 1-4 and Tables 1-4.
